# Cardiac point-of-care ultrasound reveals unexpected, life-threatening findings in two children

**DOI:** 10.1186/s13089-020-0154-3

**Published:** 2020-02-04

**Authors:** Stephanie J. Doniger, Nicholas Ng

**Affiliations:** 1 Department of Pediatric Emergency Medicine, CHOC Children’s Hospital of Orange County, Orange, CA USA; 2 Department of Pediatric Critical Care, Morgan Stanley Children’s Hospital of New York-Presbyterian, New York, NY USA

**Keywords:** Focused cardiac ultrasound, Point-of-care ultrasound, Pediatric emergency medicine, Pericardial effusion, Tamponade, Anterior mediastinal mass

## Abstract

**Background:**

The diagnosis of pericardial effusion with cardiac tamponade can at times be elusive in pediatric patients since it is relatively uncommon. Point-of-care ultrasound (POCUS) can readily be performed at the bedside to assess for the presence of a pericardial effusion, tamponade, and can occasionally yield unexpected results.

**Case presentation:**

Two cases where POCUS unexpectedly identified pericardial effusions, with one patient who also had an anterior mediastinal mass.

**Conclusions:**

Though underutilized, cardiac POCUS in children can be immediately life-saving and drastically change the clinical management at the patient’s bedside.

## Background

Point-of-care ultrasound (POCUS) is designed to answer very specific questions in real time. Additionally, it can help expeditiously make life-saving diagnoses, while avoiding unnecessary or potentially costly testing or imaging [1]. An important POCUS application in the emergency and critical care settings is the focused cardiac examination. The primary goal of focused cardiac POCUS is to identify pericardial effusions, tamponade, and asystole. Overall, the use of cardiac POCUS is underutilized in the pediatric patient population. This is presumably because the overall incidence of acquired (non-congenital) cardiac pathology in pediatric populations is quite low when compared with adult patient populations. Nonetheless, the identification of cardiac pathology in children can be immediately life-saving.

There exists a tremendous body of literature that supports the use of cardiac POCUS in diagnosing pericardial effusions in adult patient populations. Mandavia et al. [2] studied bedside cardiac POCUS performed by emergency medicine physicians in patients at high risk for pericardial effusions, with an overall accuracy of 98%, sensitivity of 96% and specificity of 98%. In patients with penetrating chest trauma and traumatic pericardial effusions, Plummer et al. [3] showed that cardiac POCUS improved outcomes when compared to those who did not receive cardiac POCUS, with shorter time to diagnosis (15.5 versus 42.4 min) and better overall survival (100% versus 57%).

There is limited literature on focused cardiac POCUS in pediatric patient populations. The majority of studies involving cardiac POCUS to identify pericardial effusions and tamponade in the pediatric ED setting are limited to case reports [4, 5]. However, in a prospective observational study of 70 pediatric emergency department patients, the overall sensitivity and specificity of cardiac POCUS in detecting diminished LV function, pericardial effusions, and abnormal IVC collapsibility, when compared with comprehensive echocardiogram was 95% (95% CI 82–99%) and 83% (95% CI 64–93%), respectively [6].

Cardiac tamponade clinically presents as Beck’s triad: jugular venous distension, muffled heart tones and hypotension [7]. However, only one-third of patients with tamponade will have all three features and 10% will not have any of them. POCUS can readily identify both pericardial effusions and sonographic tamponade [8]. The diagnosis of sonographic cardiac tamponade can be made before a patient becomes hypotensive or has clinical signs of tamponade. Signs of sonographic tamponade include a circumferential pericardial effusion, accompanied by poor filling and/or diastolic collapse of the right ventricle (“scalloping”) due to increased intrapericardial pressure, which results in reduction of stroke volume and cardiac output [9, 10].

Pericardiocentesis is the definitive treatment for cardiac tamponade. Ultrasound-guidance for pericardiocentesis has been shown to improve success rates and decrease complications when compared with blind attempts [11]. Though it is a rare condition in the pediatric population, pericardiocentesis may be indicated after blunt or penetrating trauma, or after cardiac catheterization or cardiac surgery [12]. Tsang et al. showed that those pediatric patients who had ultrasound-guided pericardiocentesis, had a 99% success rate, with 93% on the first attempt, and 1% major and 3% minor complication rates [13].

The identification of cardiac masses is not the primary goal of POCUS. However, this may be an unexpected or incidental finding. Alternatively, in a child presenting with a widened mediastinum on chest radiograph, a focused cardiac POCUS may be expeditiously performed to evaluate for the presence of a mass at the bedside.

## Case presentations

The following are two cases where POCUS unexpectedly identified pericardial effusions, with one patient who also had an anterior mediastinal mass.

### Case 1

A 12-year-old male was admitted to the pediatric inpatient unit for treatment of pneumonia. After being in the hospital for 7 days without improvement of the pneumonia, the patient acutely developed progressively worsening chest pain, shortness of breath, and diaphoresis. The pediatric intensive care team was consulted overnight for evaluation of the patient, and the patient was noted to be ill appearing and sitting in an upright “tripod” position. He was afebrile with a respiratory rate (RR) 38, heart rate (HR) 122, blood pressure (BP) 118/70, pulse oximetry 95% on simple facemask. No electrocardiogram (EKG) had been performed during his hospital admission. A Pediatric Emergency Medicine (PEM) fellow with extensive POCUS training performed a POCUS examination, to evaluate the progression of the previously diagnosed pneumonia as the likely etiology of the patient’s acute decompensation.

**Fig. 1:**
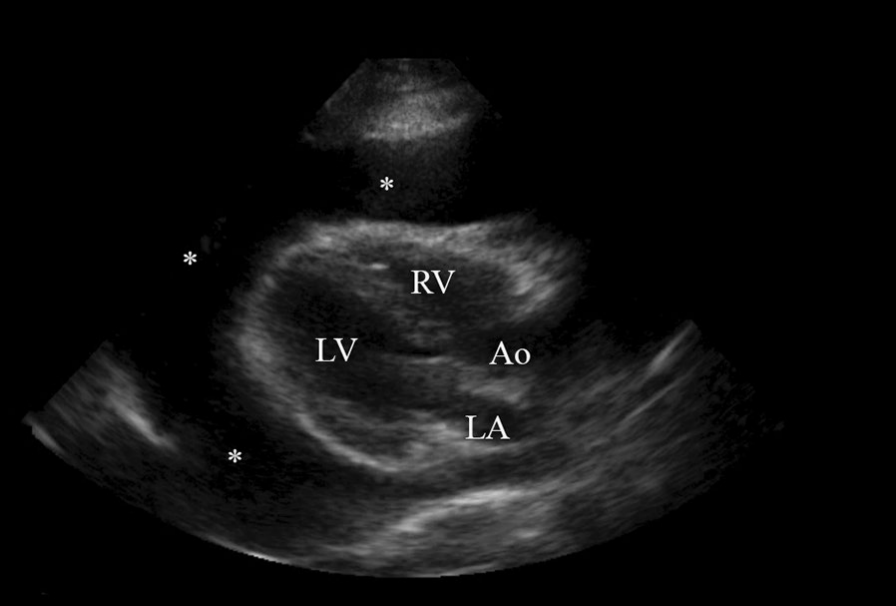
POCUS, Case 1. Parasternal long-axis view of pericardial tamponade with a large circumferential pericardial effusion (*). The classic “swinging heart” was visualized in real-time ultrasonography. For orientation, the “emergency medicine” orientation was used for scanning. Structures visualized are: the left ventricle (LV), right ventricle (RV), left atrium (LA) and aortic outflow tract (Ao)

A lung ultrasound was initially performed to evaluate the progression of the previously diagnosed pneumonia. However, the normal lung artifacts were obliterated by a large anechoic structure. Therefore, a focused cardiac POCUS was performed, which revealed a large circumferential pericardial effusion. This heart exhibited a “swinging” movement within the large effusion (Fig. 1), and diastolic right ventricular collapse. The inferior vena cava (IVC) was visualized as being dilated without respiratory variation. As a result of these findings, the diagnosis of cardiac tamponade was made, and a pediatric cardiologist was immediately consulted. The pediatric cardiologist performed an emergent ultrasound-guided pericardiocentesis, yielding one liter of serosanguinous fluid.

### Case 2

**Fig. 2:**
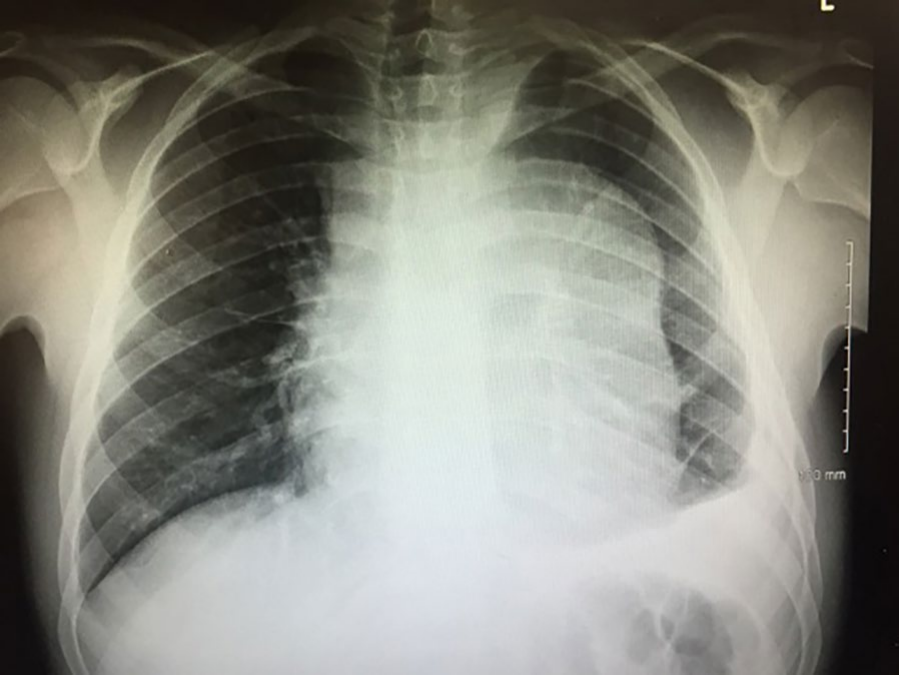
Chest radiograph, Case 2. This AP chest radiograph exhibits a widened mediastinum, suggestive of a mediastinal mass

A 17-year-old male, presented to the Pediatric ED with a 4-month history of intermittent chest pain and progressively worsening shortness of breath for the past month. He was referred from a local urgent care facility with an “abnormal chest X-ray” (Fig. 2). Of note, the patient also had an “abnormal chest X-ray” 6 months prior, but was lost to follow-up. This prior X-ray was not available for review. On examination, he was diaphoretic, sitting upright in mild respiratory distress. His vital signs were: 98 F, RR 32, HR 102, BP 110/60, pulse oximetry 95% on room air. A PEM physician with extensive experience in POCUS performed the focused cardiac POCUS.

**Fig. 3:**
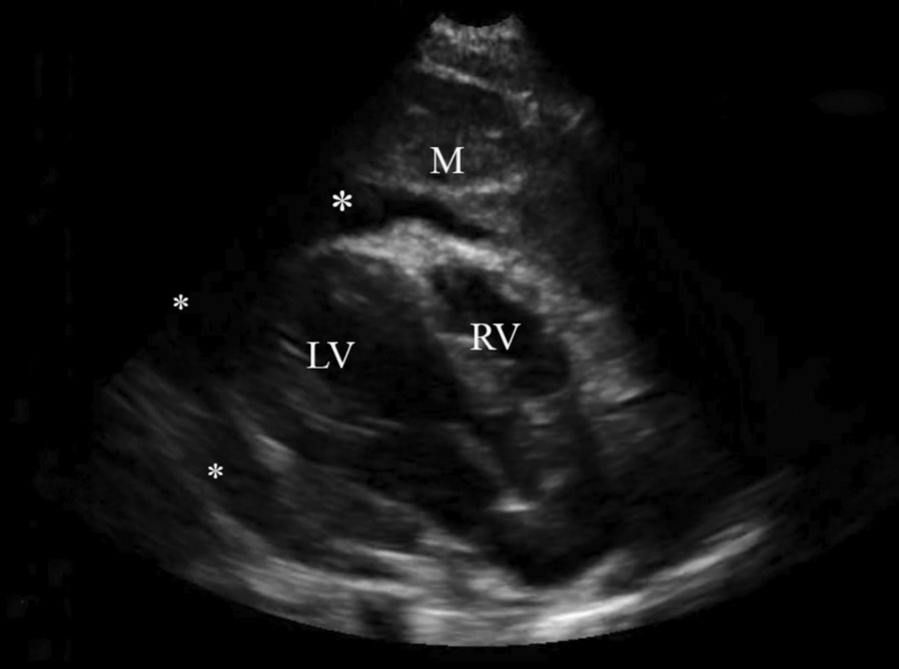
POCUS, Case 2. Parasternal long-axis view, revealing a circumferential pericardial effusion (*) and an anterior mediastinal mass (M). For orientation, the left ventricle (LV) and right ventricle (RV) can be visualized in this view

The focused cardiac POCUS showed a large mediastinal mass with a circumferential pericardial effusion, diastolic right ventricle collapse and a small left pleural effusion (Fig. 3). These findings were consistent with sonographic tamponade, which was unexpected, given that the patient did not yet exhibit clinical tamponade (BP and HR were noted to be within normal limits for age). Cardiothoracic surgery was consulted, and the patient was admitted to the cardiac intensive care unit. Due to the POCUS findings, computerized tomography (CT) of the chest was postponed due to the risk of cardiovascular collapse with supine positioning. Had there not been the information from the POCUS, a chest CT would have been performed. Ultimately a pericardiocentesis was performed by the cardiothoracic surgeons in the operating room. Surgical pathology revealed a B-cell lymphoma.

## Conclusions

There remains a tremendous potential to study the utilization of cardiac POCUS in pediatric patients in the acute care setting. Overall, cardiac POCUS may be a useful adjunct to the clinical examination when radiologic and laboratory studies can be unreliable, non-specific, and not always timely. However, it is important to note that since it is a focused examination, it is not meant to replace comprehensive echocardiography [9]. The use of focused cardiac POCUS for suspected pericardial effusions, tamponade, and mediastinal masses presents an opportunity for pediatric emergency medicine, cardiology, and critical care services to collaborate and establish best practices for these, at times, elusive diagnoses.

Though relatively uncommon, it is important to consider cardiac etiologies in the differential diagnosis of a pediatric patient presenting with shortness of breath. The above cases illustrate how POCUS can help identify pericardial effusions, tamponade, and mediastinal masses when diagnostic clarity may be lacking.

## Data Availability

Not applicable.

## Abbreviations

Ao: aortic outflow tract
BP: blood pressure
CXR: chest X-ray, radiograph
CT: computerized tomography
EKG: electrocardiogram
HR: heart rate
IVC: inferior vena cava
LA: left atrium
LV: left ventricle
PEM: pediatric emergency medicine
PICU: pediatric intensive care unit
POCUS: point-of-care ultrasound
RR: respiratory rate
RV: right ventricle
US: ultrasound
